# Antisense oligonucleotides and their applications in rare neurological diseases

**DOI:** 10.3389/fnins.2024.1414658

**Published:** 2024-09-23

**Authors:** Simon McDowall, May Aung-Htut, Steve Wilton, Dunhui Li

**Affiliations:** ^1^School of Human Sciences, The University of Western Australia, Crawley, WA, Australia; ^2^Perron Institute for Neurological and Translational Science, The University of Western Australia, Nedlands, WA, Australia; ^3^Centre for Molecular Medicine and Innovative Therapeutics, Health Futures Institute, Murdoch University, Murdoch, WA, Australia

**Keywords:** rare diseases, antisense oligonucleotides, treatments, therapeutics rare disease, oligonucleotide, precision therapeutics

## Abstract

Rare diseases affect almost 500 million people globally, predominantly impacting children and often leading to significantly impaired quality of life and high treatment costs. While significant contributions have been made to develop effective treatments for those with rare diseases, more rapid drug discovery strategies are needed. Therapeutic antisense oligonucleotides can modulate target gene expression with high specificity through various mechanisms determined by base sequences and chemical modifications; and have shown efficacy in clinical trials for a few rare neurological conditions. Therefore, this review will focus on the applications of antisense oligonucleotides, in particular splice-switching antisense oligomers as promising therapeutics for rare neurological diseases, with key examples of Duchenne muscular dystrophy and spinal muscular atrophy. Challenges and future perspectives in developing antisense therapeutics for rare conditions including target discovery, antisense chemical modifications, animal models for therapeutic validations, and clinical trial designs will also be briefly discussed.

## 1 Introduction

Although the definition of a rare disease (sometimes called an “orphan disease”) varies in different countries, an individual disease is typically defined as rare when the number of affected people falls in the range of < 0.07%. Despite having a low prevalence when considered individually, rare diseases collectively are estimated to affect more than 500 million people worldwide, and almost half of these rare diseases are neurological (Nguengang Wakap et al., 2020; The Lancet Neurology, 2022). There has been extraordinary progress in the design of drugs to treat rare diseases with over two hundred new therapies developed over the last few years, achieving the goal set by the International Rare Diseases Research Consortium (Abbott, 2011). For rare conditions resulting from enzyme deficiencies, enzyme replacement therapy is the major treatment strategy. Examples include Brineura^®^ for neuronal ceroid lipofuscinosis type 2, Fabrazyme^®^ for Fabry’s disease, and Lumizyme^®^ for Pompe’s disease. However, heterogeneity in the genetic background of patients and the subsequent clinical severity, including the rate of disease progression, often results in variable patient responsiveness to these treatments (Concolino et al., 2018). The majority of rare diseases still lack effective and approved treatments, with the most common impediment being the lack of a definitive genetic diagnosis (Blin et al., 2020). Traditional genetic analysis is best suited to diagnose monogenic conditions, such as Duchenne muscular dystrophy (Duchenne MD), resulting from *dystrophin* mutations, or cystic fibrosis, which is caused by cystic fibrosis transmembrane conductance regulator (*CFTR)* mutations (Brewington and Clancy, 2016; Okubo et al., 2016). Following clinicians providing a clinical diagnosis and selecting the likely causative genes for further analysis, molecular genetic diagnostic methods can be supported by family history and inheritance patterns. Furthermore, many rare disorders do not present with sufficiently distinct clinical presentations, making a definitive diagnosis challenging. Development of whole-genome sequencing technology has allowed for the potential comprehensive and highly effective diagnosis of individuals with rare diseases, yet the widespread implementation of such technology is hampered by high costs of sequencing, ethical concerns, and resource-intensive changes to the workflow of genetic diagnostic laboratories (Chaubey et al., 2020; Schobers et al., 2024). Therefore, accurate diagnosis, identification of appropriate patients, and optimal timing for treatment are crucial for the development and evaluation of effective therapeutics for rare neurological conditions (Papaioannou et al., 2023).

## 2 Potential drug discovery strategies for rare disorders

Orphan drug development has rapidly accelerated since 2011, coinciding with the goal set by the International Rare Diseases Research Consortium to have 1,000 new therapies approved for rare diseases by 2027 (Austin et al., 2018). Reaching this milestone will require dramatically more efficient processes for drug discovery and development. As reviewed in a recent paper (Papaioannou et al., 2023), small molecules, antibodies, cell, and gene therapies are currently the main focus of research into treatment of rare diseases. However, there are pros and cons of each therapeutic modality. Industry has traditionally focused on small molecules, which can be produced at reasonable costs and have the ability to target multiple tissues, keeping them at the forefront of orphan drug development (Singh et al., 2020; Tambuyzer et al., 2020). Despite this, a considerable amount of time is required to find an ideal therapeutic candidate that displays optimal pharmacological and pharmacokinetic characteristics while maintaining an acceptable safety and tolerability profile. New technologies including high-throughput and high-content screening of small molecules are advancing the discovery and design of novel bioactive molecules (Markossian et al., 2018; Bellomo et al., 2017). However, the slow rate at which small molecule therapies reach the clinic is still a concern for orphan drug development (Mifsud and Cranswick, 2022).

Drug repurposing informed by a particular compound’s mechanism of action, or accidental observations in some cases, can expand the role of existing drugs, thereby circumventing the need for novel discovery (Bellomo et al., 2017). For example, Lonafarnib, a molecule shown to reduce hepatitis D virus levels in the blood, has been reutilized for the treatment of Progeria and progeroid laminopathies (Baines et al., 2011; Cappato et al., 2018; Gordon et al., 2018). To date, approximately 20% of the 127 orphan drugs approved in the European Union are repurposed (Scherman and Fetro, 2020), although treatment discovery remains difficult for rare conditions. This is especially true for disorders with complex or poorly characterized disease mechanisms and inadequate or absent natural history studies. Drug screening is often relatively expensive and would be particularly challenging for 1–2 patients. Therefore, developing novel therapies targeting the disease mechanism and corresponding molecular pathways is logical, and could provide hope for those affected by rare conditions.

Oligonucleotide therapies entered the clinic over 20 years ago, beginning in 1998 with the US Food and Drug Administration (FDA) approval of Fomivirsen, an antisense oligonucleotide (ASO) for the treatment of cytomegalovirus retinitis in immunocompromised patients (Vitravene Study Group, 2002). Depending on the chemical composition and/or structure of oligonucleotides, there are a variety of mechanisms by which these molecules can modify gene expression. To date, 16 antisense treatments including ASOs and small interfering RNAs have been given FDA or EMA approvals for the treatment of genetic disorders (Table 1; Stein and Castanotto, 2017; Yin and Rogge, 2019; Heo, 2020).

**TABLE 1:** List of antisense oligonucleotide drugs targeting mRNAs and pre-mRNAs approved by the US Food and Drug Administration (FDA) or the European Medicines Agency (EMA).

Drug name	FDA Approval	EMA Approval	Rare disease	Oligonucleotide type	Mechanisms	Companies involved
Fomivirsen	1998 (Withdrawn)	1999 (Withdrawn)	Cytomegalovirus retinitis	Antisense oligonucleotide	RNase H	Ionis Pharmaceuticals, Novartis
Mipomersen	2013 (Withdrawn)		Homozygous Familial Hypercholesterolemia	Antisense oligonucleotide	RNase H	Ionis Pharmaceuticals, Genzyme, Kastle Therapeutics
Eteplirsen	2016 (Marketed)		Duchenne muscular dystrophy	Antisense oligonucleotide	Exon skipping	Sarepta Therapeutics
Nusinersen	2016 (Marketed)	2017 (Marketed)	Spinal muscular atrophy	Antisense oligonucleotide	Splicing modulation/exon inclusion	Ionis Pharmaceuticals, Biogen
Inotersen	2018 (Marketed)	2018 (Marketed)	Familial Amyloid Neuropathies	Antisense oligonucleotide	RNase H	Ionis Pharmaceuticals, Akcea Therapeutics
Patisiran	2018 (Marketed)	2018 (Marketed)	Familial Amyloid Neuropathies	siRNA	RNAi	Alnylam Pharmaceuticals
Milasen	2018 (*n* = 1 treatment)		DNA mutation associated with *CLN7* gene, resulting in Batten disease in a young girl, Mila Makovec	Antisense oligonucleotide	Splicing modulation	Boston Children’s Hospital
Volanesorsen		2019 (Marketed)	Familial chylomicronemia syndrome	Antisense oligonucleotide	Exon skipping	Ionis Pharmaceuticals
Givosiran	2019 (Marketed)	2020 (Marketed)	Acute Heptatic Porphyria	siRNA	RNAi	Alnylam Pharmaceuticals
Golodirsen	2019 (Marketed)		Duchenne muscular dystrophy	Antisense oligonucleotide	Exon skipping	Sarepta Pharmaceuticals
Viltolarsen	2020 (Marketed)		Duchenne muscular dystrophy	Antisense oligonucleotide	Exon skipping	NS Pharma, Inc
Lumasiran	2020 (Marketed)	2020 (Marketed)	Hyperoxaluria type 1	siRNA	RNAi	Alnylam Pharmaceuticals
Inclisiran	2021 (Marketed)	2021 (Marketed)	Familial hypercholesterolemia	siRNA	RNAi	The Medicines Company, Novartis
Casimersen	2021 (Marketed)		Duchenne muscular dystrophy	Antisense oligonucleotide	Exon skipping	Sarepta Therapeutics
Vutrisiran	2022 (Marketed)		Hereditary transthyretin amyloidosis	Antisense oligonucleotide	RNase H	Alnylam Pharmaceuticals
Tofersen	2023 (Marketed)		Amyotrophic lateral sclerosis	Antisense oligonucleotide	RNase H	Ionis Pharmaceuticals

RNAi, RNA interference; siRNA, small interfering RNA.

**FIGURE 1:**
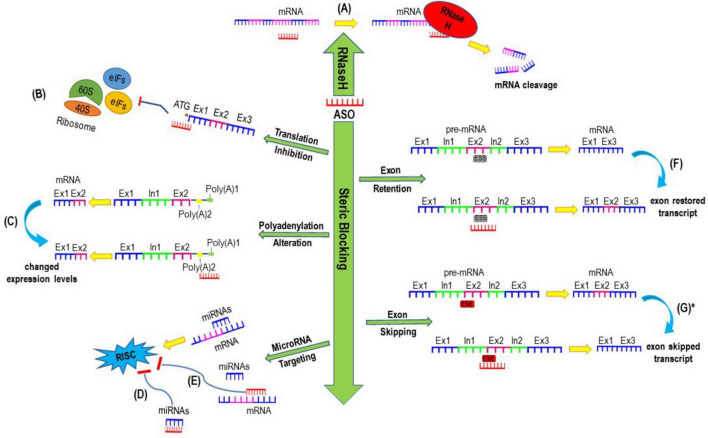
Examples of mechanisms of actions induced by antisense oligonucleotides. **(A)** Antisense oligonucleotide (ASO) mediated mRNA cleavage and degradation through inducing RNase H; **(B)** Translation inhibition mediated by ASO blocking the translation initiation codon (ATG) which subsequently interferes ribosomal subunits from translating mRNAs to proteins. **(C)** ASOs designed to sterically block alternative polyadenylation sites which could result in either stable or unstable transcript, leading to increased or decreased expression of protein of interest. **(D)** ASOs can be designed to either bind to microRNAs or **(E)** sterically block the binding site of any microRNAs to inhibit the activities of microRNA and prevent the formation and activities of RNA-induced silencing complex (RISC), thereby increase the expression of targeted gene. **(F)** ASOs mediated exon retention after annealing to intronic splice silencer (ISS) **(G)** ASOs targeting exon splicing enhancers (ESE) can induce exon skipping to generate a shorter exon skipped transcript. *Exon-skipped shorter transcript can either be translated into a shorter functional protein if the reading frame is restored or be degraded through non-sense mediated decay if the reading frame is shifted and a premature termination codon is induced.

ASOs are short synthetic nucleic acid analogues that anneal to reverse complementary sequences through Watson-Crick base pairing (Chakravarthy et al., 2017). With appropriate design, ASOs can alter the expression of targeted genes through a variety of mechanisms of actions as seen in Figure 1, which includes modulating splicing, suppressing translation initiation, altering polyadenylation, recruiting RNaseH enzyme to cleave targeted transcripts, and inhibiting microRNA activities (Rinaldi and Wood, 2018; Crooke, 2017). There are other ASO mechanisms of action including but not limited to modulating translation by blocking functional *cis*-acting elements in the 5′-untranslated region and inhibiting nonsense-mediated decay as reviewed in recent articles (Crooke et al., 2021a; Crooke et al., 2021b). Since approximately 80% of rare diseases arise from a genetic alteration in single genes (Groft et al., 2021), ASOs can be developed to target pathological mutations and bring disease modifying therapeutic benefits to patients with these conditions (Tambuyzer et al., 2020; Han et al., 2022). However, it is important to carefully consider the use of ASOs in lieu of gene editing strategies such as CRISPR-CasRx, base editing and prime editing, enzyme replacement, small molecule therapy or gene replacement strategies (Issa et al., 2023; Li et al., 2022). We direct readers to a recently published excellent review paper that highlights several crucial factors to determine if a rare genetic disorder can be treated using ASO therapeutics (Lauffer et al., 2024). Such considerations include which ASO chemistry to use, tissue targets, and whether the benefits of the treatment will outweigh the burden and risks of repeated administration and off-target effects. Global guidelines and recommendations for individualized treatment of rare genetic disorders through ASO-therapeutics are currently being developed, which will allow for the standardization of which diseases and people are eligible to be treated using this strategy (Aartsma-Rus et al., 2023a; Aartsma-Rus et al., 2023b).

## 3 Therapeutic antisense oligonucleotides

Sequence-specific inhibition of Rous sarcoma virus production in chick embryo fibroblasts using a chemically synthesized ASO was first reported in 1987 (Zamecnik and Stephenson, 1978). Numerous applications of antisense therapeutics have since been investigated, with some demonstrating therapeutic potentials for numerous inherited and acquired disorders (Mani et al., 1999; Moon et al., 2000; Engelhard, 1998; Singh et al., 2006; Collotta et al., 2023). The FDA authorization of Fomivirsen, for the treatment of cytomegalovirus retinitis in 1998, was the first approval for an antisense drug. However, it took nearly two decades for additional antisense compounds including Eteplirsen, Nusinersen, Inotersen, Volanesorsen, Golodirsen, Viltolarsen, Casimersen, and Tofersen to be clinically approved for several different indications. Bottlenecks impeded the early stages of ASO drug development, with an initial limitation being the susceptibility of oligonucleotides to endo- and exonuclease degradation. To combat this, chemical modifications to the bases and backbone were introduced (Agrawal, 1999). Consequently, evolving ASO chemistries greatly improved the stability of these molecules, as well as the consistency of large-scale synthesis and cellular uptake (Kim, 2023).

### 3.1 Antisense oligonucleotide chemistries

The natural phosphodiester bond found in RNA and DNA strands is vulnerable to endogenous nuclease activity, while chemical modifications to this backbone chemistry make ASOs less likely to be degraded. Replacing the non-bridging oxygen atom in the phosphodiester backbone with a sulfur atom, creates a phosphorothioate linkage with enhanced resistance to degradation (Wilton et al., 2015; De Clercq et al., 1969). The creation of a phosphorothioate linkage is the first generation of ASO modifications improving the practicality of ASO synthesis (Eckstein, 2014). This backbone chemistry is now widely used for *in vitro* ASO evaluation and is increasingly being studied *in vivo*. Moreover, enhanced RNA/DNA binding affinity and additional nuclease degradation resistance of ASOs can be achieved by altering the ribose sugar at the 2′ position (McKay et al., 1999). These sugar modifications mainly include 2′-O-methyl (2′-OMe) (Majlessi et al., 1998), 2′-O-methoxy-ethyl (2′-MOE) (Geary et al., 2001), and 2′-fluoro (2′-F) (Kawasaki et al., 1993), and confer antisense compounds with a higher binding affinity to the recognition sequence when combined with a phosphorothioate backbone (Freund et al., 2019). The induction of conformationally constrained nucleotides further increases the target affinity and ASO biostability (Murray et al., 2012). These modifications include inducing an additional methylene bridge connecting 2′ oxygen and 4′ carbon in the ribose moiety to create a locked nucleic acid (LNA) (Veedu and Wengel, 2009), the addition of a 2′, 4′-constrained ethyl group to the ribose sugar to produce a constrained ethyl (cEt) (Seth et al., 2008), and adding carbon-atoms between the 5′ carbon and 3′ carbon of natural DNA to generate tricyclo-DNA (tcDNA) (Goyenvalle et al., 2015). tcDNA ASOs were reported to have the ability to cross the blood-brain barrier, which may be a promising systemic alternative for the treatment of central nervous system disorders (Robin et al., 2017).

Modifications on both the sugar moiety and backbone are further refinements in ASO chemistries that offer much greater nuclease resistance, enhanced target annealing, and allow gene expression to be influenced through different mechanisms. Such modifications include peptide nucleic acid (PNA) and phosphorodiamidate morpholino oligomers (PMOs), generated by replacing the sugar-phosphate backbone with non-ribose polyamide linkages (Schultz and Gryaznov, 1996), or substituting the ribose base with a morpholino ring (Norris and Streit, 2014), respectively. These particular alterations result in an uncharged compound, and the substantial variation from the natural phosphodiester backbone greatly improves nuclease resistance. Due to their neutral charge, these molecules are less likely to interact with plasma proteins, while still maintaining strong binding to complementary nucleic acids. An excellent safety profile and efficacy data have been reported from clinical trials after systemic administrations of different PMOs (Mendell et al., 2016; Vasterling et al., 2023). However, these uncharged oligomers do have some limitations, including rapid clearance from circulation through kidney and relatively poor cellular uptake (Moulton and Moulton, 2010). One approach to address this issue is the conjugation of cell-penetrating peptides to the PMOs to increase cellular uptake. Among these peptides, Tat-derived arginine-rich cell-penetrating peptides were investigated and show some tissue specificity in mouse models (Moulton et al., 2007; Jearawiriyapaisarn et al., 2008; Echigoya et al., 2017). While highly efficient in rodent models, these peptide-conjugated PMOs have been found to induce severe dose-dependent toxic effects in non-human primates (Moulton and Moulton, 2010; Haque and Yokota, 2023) which the toxicity issues might be mitigated by trying different types or compositions of amino acids in a peptide (Reveret et al., 2023). Having demonstrated proof-of-concept, studies in developing a safe and effective cell penetrating peptide have entered the clinic. A Phase 2 trial of SRP-5051, a conjugate of a proprietary peptide with Eteplirsen, a PMO designed to skip exon 51 from the *DMD* gene to re-frame the dystrophin mRNA in amenable patients, showed substantially better efficacy, including higher increases in dystrophin protein and exon skipping with clinically manageable hypomagnesemia, as reported by Sarepta Therapeutics (Sorrentino, 2024).

Additional ASO modifications, including exonuclease resistant anhydrohexitol nucleic acid (HNA) (Le et al., 2016a), human serum stable altritol nucleic acid (ANA) (Le et al., 2016b), thermostable twisted intercalating nucleic acid (TINA) (Le et al., 2016b), nucleobase modification C5-phenyltriazole DNA (Le et al., 2017) and thiomorpholinos (Le et al., 2022) have shown high exon skipping efficacies *in vitro*. However, these chemistries may receive additional scrutiny because a higher oligomer concentration might be required to induce efficient therapeutic effects. As the phosphorothioate substitution changes the achiral phosphodiester linkage into a chiral phosphorothioate center, all phosphorothioate backbone ASOs consist of thousands of stereoisomers, resulting from the inability to control the configuration of phosphorothioate installation (Crooke and Geary, 2013). Stereopure ASOs may be produced by controlling the achiral phosphorothioate linkage and were proposed as one of the latest breakthroughs in ASO synthesis by Wave Life Sciences (Iwamoto et al., 2017). These ASOs were reported to offer increased efficacy, safety and persistence of the oligomer *in* vivo (Iwamoto et al., 2017). Although Wave Life Sciences promised a great deal for the *DMD* exon skipping field, their Duchenne MD program was first halted in December 2019 and soon after discontinued completely (Markati et al., 2021).

### 3.2 Off-target effects and toxicities

Chemical modifications have endowed antisense oligomers with better solubility and stronger resistance to endo- and exonucleases; however, altering the base-pair sequence or backbone of ASOs can result in severe toxic consequences (Stanton et al., 2012; Hagedorn et al., 2022). Antisense oligomer-related toxicities can be mediated through different mechanisms. Hybridisation-dependent toxicities may arise from off-target annealing to an unintended RNA that features some homology, disrupting the expression of other genes. These particular off-target effects are more commonly seen in the RNase H activating ASOs (Dieckmann et al., 2018). In contrast, for steric blocking ASOs that are highly dependent on sequence position in the target RNA, annealing to unintended RNA is rare, even when there is a perfect match between the steric blocking ASOs and unintended RNA (Obad et al., 2011; Kamola et al., 2017). A study by Scharner et al. (2020) that aimed to investigate sequence-specific off-target effects of two overlapping splice-switching ASOs investigated 108 potential ASOs targets and found that mixed chemistry ASO (DNA/2′-MOE or DNA/ctE hybrids) were found to significantly reduce mismatch base pairing compared to an 2′-MOE ASO with the same sequence. Significantly, it was reported that shorter ASOs had less off-target effects, most likely due to having higher binding specificity for the target RNA sequences, and are more effective at penetrating into cells and tissues (Derbis et al., 2021). Although investigations on longer ASOs have not been completed, it was found that increased ASO length may improve the efficiency of exon-skipping for some transcripts compared to others (Harding et al., 2007; Heemskerk et al., 2009), suggesting that further studies are needed to characterise which target would benefit most from modifications to ASO length.

ASO-interaction with intra- and extracellular proteins, or the accumulation of antisense compounds may be hybridisation-independent (Frazier, 2015; Collotta et al., 2023). Due to the polyanionic nature of phosphorothioate ASOs, these oligomers can bind to many serum proteins including heparin-binding EGF-like growth factors, as well as intracellular and extracellular receptors, causing renal or hepatic toxicities. For instance, the hepatoxicity of LNA modified ASOs is speculated to result from the aptameric binding of LNAs to hepatic intracellular proteins (Kakiuchi-Kiyota et al., 2014; Burdick et al., 2014). Furthermore, when LNA ASOs contain TGC and TCC motifs in a 3-8-3 gapmer design, they exhibit a higher propensity to bind to mouse liver proteins and increase hepatotoxicity (Burdick et al., 2014). LNA-associated nephrotoxicity is suspected to arise from the binding of these compounds to proteins in proximal tubule cells. However, renal toxicity is more likely to result from the build-up of ASOs within proximal tubule lysosomes (Henry et al., 2007), with evidence showing tubular necrosis and ASO accumulation in a kidney biopsy from the first human trial of an LNA ASO (van Poelgeest et al., 2013). Moreover, phosphorothioate ASOs bind more tightly and readily to membrane-less protein structures such as nuclear paraspeckles compared to ASOs with the natural phosphodiester backbone (Bailey et al., 2017; Liang et al., 2021). Such ASOs targeting key components of paraspeckles, such as the long non-coding RNA, *NEAT1*, may lead to neuronal stress, and a reduction in the amount of ASO available to bind to target transcript (Shen et al., 2014; Sekar et al., 2022).

ASOs may also cause toxicities by activating proinflammation and stimulating immune systems. The presence of unmethylated cytosine-phosphate-guanosine (CpG) dinucleotides in oligodeoxynucleotides can be recognised by the Toll-like receptor 9, thereby stimulating the immune system (Hanagata, 2012). Evidence suggests that the phosphorothioate backbone has innate immunostimulatory activity and can also activate the complement system (Hartmann et al., 1996; Henry et al., 2002; Henry et al., 1997). Significant upregulation of immune system associated genes was observed in mouse brains after intracerebroventricular administration of phosphorothioate, 2′-OMe modified ASOs (Toonen et al., 2018). In monkeys, chronic intravenous infusion of an ASO featuring a 2′-O-methoxyethyl modification on the phosphorothioate backbone, correlated with increased plasma concentration of complement split products including Bb, C3a and C5a, thus indicating activation of the alternative pathway of the complement system (Henry et al., 1997; Shen et al., 2016). However, oligonucleotide-induced complement activation was absent in human and dog studies (Henry et al., 2014), likely due to insufficient plasma concentration being achieved in those clinical trials.

Additional ASO-related toxicities such as thrombocytopenia, have been noted occasionally in clinical trials (Crooke, 2007; Frazier, 2015). Consequently, these harmful side-effects are a major factor impeding ASO therapeutic development. Carefully testing ASOs in at least two animal species and addressing these toxicities prior to human studies, may minimise their emergence in clinical trials. Moreover, selecting safer compounds with maximum potency by rational ASO design may help advance antisense compounds from the bench to bedside faster.

## 4 Splice-switching antisense oligonucleotides and potential applications in rare neurological conditions

Splice modifying ASOs can be designed to induce exon skipping by targeting acceptor or donor splice sites, as well as splice enhancer elements, or to promote exon retention by blocking splicing silencers. Additionally, splice-modifying ASOs can suppress abnormal splicing by masking cryptic splice motifs (Aung-Htut et al., 2019; Havens and Hastings, 2016). Although it would seem highly probable that splicing can be altered by steric blocking of the crucial acceptor or donor splice sites, not all ASOs directed towards these motifs alter splicing (Mitrpant et al., 2009). Indeed, most ASOs targeting these sites are ineffective. For instance, 2′-OMe ASOs targeting the mouse *Dmd* exon 23 acceptor site had no effect on pre-mRNA processing, whereas targeting the donor site efficiently induced skipping of that exon. Comparatively, ASOs targeting the equivalent human dystrophin pre-mRNA donor site had variable effects on splicing (Mitrpant et al., 2009), suggesting the involvement of other factors.

The first report of *in vitro* ASO-mediated splicing correction occurred in 1993, when antisense compounds were designed to restore intronic β-globin mutations causing β-thalassemia in cell-free nuclear extracts (Dominski and Kole, 1993). Since then, splice-switching ASOs have drawn considerable attention as therapeutics for a number of diseases, including Duchenne MD (Cirak et al., 2011; Matsuo, 1996) and spinal muscular atrophy (SMA) (Edinoff et al., 2021). The strategy of designing splice-switching ASOs to induce exon skipping as a treatment for Duchenne MD is based on the Monaco reading frame rule (Monaco et al., 1988) and the clinical genotype-phenotype correlations between Duchenne and Becker MD (Wilton et al., 2007), where deletion of particular in-frame exons or exon blocks leads to milder clinical symptoms and slower disease progression (Muntoni et al., 2003). The dispensable exons may be regarded as non-essential, as their excision from the transcripts generates internally truncated proteins with near-normal functions in some combinations. It is hypothesized that of the 562,164 exons reported in the human genome, additional exons may be conditionally dispensable on a case-by-case basis, and can be potentially removed as a therapeutic strategy for many diseases (Piovesan et al., 2019; Li et al., 2018).

### 4.1 The reading frame rule in DMD, non-essential exons and the exon-skipping strategy for Duchenne muscular dystrophy

Monaco’s “reading frame hypothesis” generally explains the more obvious genotype-phenotype correlations in Duchenne and Becker MD (Monaco et al., 1988). In most cases, genomic deletions that shift the *DMD* translational open reading frame result in expression of prematurely truncated, non-functional dystrophin and subsequently, a severe Duchenne MD phenotype (Van Ruiten et al., 2017). Conversely, genomic deletions that do not disrupt the reading frame generally give rise to shorter but semi-functional protein isoforms and the milder Becker MD phenotype (Monaco et al., 1988), with ambulation retained until at least age 16 or older (Flanigan, 2014). In-frame deletions of exons in the *DMD* major deletion hotspot region (exons 45 to 55), within the central rod domain, are generally associated with a milder disease course (Harper et al., 2002; Aartsma-Rus et al., 2016). This has been observed for individuals with exon 45–48, 45–51 or 45–55 deletions, where such mutations lead to internally truncated dystrophin isoforms that retained most of the full-length dystrophin functionality (Nicolas et al., 2015; Taglia et al., 2015). Comparatively, patients with in-frame deletions of exons 10–44, 13–40, 3–41, or 8–50 exhibited a Duchenne MD phenotype (Nevo et al., 2003; Egorova et al., 2023). Deletions of 34 or more *DMD* exons are also associated with a severe disease course, regardless of the reading frame (Fanin et al., 1996), indicating a threshold effect on dystrophin size as losing too much of the coding region impedes formation of the resultant dystrophin-glycoprotein complex (Zhou et al., 2017). In addition, in-frame deletions of even a single exon can result in a severe phenotype if an out-of-frame pseudo exon at the deletion breakpoint is activated (Greer et al., 2015), or the exon(s) encode important functional regions. For example, in-frame deletion of *DMD* exon 5 deletes part of actin-binding domain 1 (encoded by exons 2–8) (Kyrychenko et al., 2017), disrupting the interaction between dystrophin and actin, leading to a Duchenne MD phenotype (Toh et al., 2016).

Clearly the nature of Becker MD dystrophin isoforms is of utmost importance. The size, nature and location of the mutation also influences disease severity. Nevertheless, it was estimated that the reading-frame rule hold for around 90% of dystrophinopathy mutations (Aartsma-Rus et al., 2006). Observations from thousands of Duchenne and Becker MD genotype-phenotype correlations have unequivocally identified non-essential exons, or exons that can be lost without abolishing protein function. Thus, strategies to skip these exons have been developed as promising personalized treatments for patients carrying amenable mutations, as seen with Eteplirsen (Mendell et al., 2013), Golodirsen (Heo, 2020), Casimersen (Shirley, 2021), and Viltolarsen (Roshmi and Yokota, 2022). Similarly, non-essential exons, or exons that could be omitted from the mature mRNA without seriously compromising its function may exist in other parts of the *DMD* gene, thus offering additional therapeutic intervention strategies. It was reported that splice-switching ASOs targeting a single exon are applicable to 64% of all Duchenne MD patients, and that ASOs designed to skip multiple exons extend therapeutic applications to ∼90% of deletion mutations, 80% of duplication mutations, and 98% of nonsense mutations (Echigoya et al., 2017).

### 4.2 Spinal muscular atrophy

Homozygous deletion of exons in the survival of motor neuron 1 gene (*SMN1)*, typically of exons 7 or 7 and 8, accounts for ∼95% of SMA cases globally, whilst intragenic mutations account for the remaining 5% (Niba et al., 2022). The loss of *SMN1*, located on chromosome 5q13.2, leads to the decreased production of the survival motor neuron (SMN) protein, which results in the gradual degeneration of the α-motor neurons in the spinal cord, manifesting as muscle weakness and loss (Mercuri et al., 2022). Furthermore, the clinical presentation of the disease is heterogeneous, and generally correlates to *SMN2* copy number (ranging from 0 to 8) where infants with a higher copy number of *SMN2* generally present with a less severe SMA phenotype (Finkel et al., 2017). Type 0 SMA, the rarest and most severe form of the disease and often results in death before six months of age, whereas Type 1 typically results in death before the age of 2. Children with Type 3 SMA are usually able to walk but may eventually become bound to wheelchair, while Type 4 SMA is a mild adult form with a normal life span (Burr and Reddivari, 2023; Singh et al., 2018). Unfortunately, most cases of SMA are Type I SMA, which accounts for approximately 45% of all cases, and is a severe form and is usually fatal within 1–2 years due to respiratory infections and difficulties.

Although all humans normally possess two or more copies of the *SMN2* gene, which also encodes an identical SMN protein, *SMN2* is unable to fully reconstitute normal SMN protein levels following *SMN1* loss (Sheng et al., 2020; Bürglen et al., 1995). This is due to a critical nucleotide base difference between the two paralogs, where *SMN1* possesses a cytosine at the 6^th^ position of exon 7, whereas *SMN2* possesses a thymine. While this nucleotide change does not change the protein that is encoded, an exonic splice enhancer is converted to a splice inhibitor motif so that exon 7 is omitted from the majority of the *SMN2* transcripts. Subsequently, exon 7 is not present in the translation of most *SMN2* mRNAs, resulting in the production of a truncated SMN protein that is readily degraded and nonfunctional (Mercuri et al., 2022; Butchbach, 2021). Since insufficient expression of functional SMN protein contributed to the disease, efforts have focused on increasing its production as an opportunity for therapy.

Thousands of targets including the 3’ splice site of exon 8, element 1, GC-rich sequence, and intronic splicing silencer-N2 and the 100th position of intron 7 in *SMN2* were investigated to increase the expression of a functional SMN protein (Collotta et al., 2023; Hua et al., 2007). It was the discovery of a critical weakness in the 5′ splice site of exon 7 in *SMN2* that paved the way for the development of a unique ASO therapeutic strategy to target the intronic splicing silencer N1 (ISS-N1) to promote *SMN2* exon 7 inclusion and produce a functional SMN protein (Singh et al., 2006). ISS-N1 as a promising therapeutic target was then independently validated in 2007 by the Krainer group in collaboration with ISIS Pharmaceuticals (now IONIS Pharmaceuticals) (Hua et al., 2007). After the selection of several ASO chemistries and the most efficacious ASO sequence designs (Hua et al., 2008), an 18-mer 2′-MOE ASO targeting ISS-N1 emerged as the best performing candidate (Hua et al., 2010; Hua et al., 2011) which demonstrated unprecedented therapeutic efficacy in the restoration of SMN protein levels and greater than 10-fold increase in the lifespan of severe SMA mice (Crawford et al., 2023; Hua et al., 2011). The significant improvements in physical abilities of SMA patients and no safety or tolerability concerns raised after 3 months treatment in clinical trials (Chiriboga et al., 2016) ultimately led to the FDA and EMA approvals of Nusinersen in 2016 and 2017 respectively. Furthermore, gaining motor function and reaching milestones after an average of 5 years of Nusinersen treatments in SMA patients, without significant side effects in up to 7 years of follow up, have demonstrated the safety, durability, and efficacy of ASOs to treat SMA and potentially other neurological diseases (Healio, 2023).

## 5 Antisense therapeutics for other rare neurological conditions

The FDA and EMA approvals of ASO therapies for Duchenne MD and SMA have offered key insights into the processes of target discovery, optimisation of therapeutic benefit through the testing of several different ASO chemistries and has shaped the pathway forward for the discovery of other ASO therapeutics. Milasen, a splice-modulating ASO drug specifically designed by Dr Timothy Yu’s team at Boston Children’s Hospital to treat a young girl suffering from Batten’s disease, a rare progressive neurological condition, has again showcased the potential of ASO as precision treatment (Kim et al., 2019). An insertion of an SVA (SINE-VNTR-Alu) retrotransposon in intron 6 of the *MFSD8* gene, which is also known as the *CLN7* gene, created a cryptic splice-acceptor site located 119 bp upstream from the SVA insertion site and led to premature translational termination of the *MSFD8* gene. After the identification of this pathogenic mutation, a 22-nucleotide ASO, Milasen, was designed with 2′-MOE modified bases on a phosphorothioate backbone to mask the cryptic splice site and force splicing back to the normal pattern. Upon administration of Milasen to the young patient, the frequency and duration of severe seizures, the major manifestations of the disease, were reduced by greater than 50% (Kim et al., 2019). Despite an extremely encouraging initial reduction in disease symptoms, the young patient succumbed to her disease some 3 years later, raising the obvious question “what would the prognosis have been had this personalized treatment have been initiated before the pathology was already well established?”.

Nevertheless, the story of Milasen has exemplified the incredible potential and feasibility of quickly developing ASO treatments for rare neurological conditions once amenable pathogenic genetic mutations are identified. The FDA approval of Tofersen in 2023 for the treatment of amyotrophic lateral sclerosis (Table 1) was another exciting moment that has demonstrated the significant potential of ASOs in the precision drug discovery. In addition, encouraging data have been reported in the field of developing ASO treatments for many other rare neurological diseases. For example the Phase 2 trial (NCT04740476) of a splice-switching ASO to increase the expression levels of *SCN1A* to treat Dravet syndrome (Han et al., 2020), and the Phase 3 trial (NCT06415344) of an ASO to manipulate the levels of *UBE-3A* as a novel therapy for treating Angelman syndrome. Many other preclinical studies have been done to investigate the therapeutic efficacies of ASOs for rare disorders including spastic paraplegia 49 (Meyyazhagan and Orlacchio, 2022), ataxia telangiectasia (Petley et al., 2022), and rare juvenile-onset Parkinson’s disease arising from PARK2 mutations (Li et al., 2020). More information on developing ASO therapies for rare neurological conditions can also be found in recent published reviews (Synofzik et al., 2022; McCauley and Bennett, 2023).

## 6 Challenges in developing antisense therapies for rare neurological diseases

The successful translations of ASO therapeutics to treat two “common” rare neurological and neuromuscular conditions have highlighted the enormous potential of this therapeutic strategy, raising hopes to extend targeted and effective treatments for many other rare diseases. However, significant challenges and obstacles still exist that impede the implementation of tailored ASO therapies to treat patients with rare or unique mutations. The rationale design of ASO strategies can be rapidly developed once an accurate identification and understanding of the genetic cause underlying a rare disease is certain; although the discovery of the most druggable target may usually take significant time and efforts as exemplified by the development of Nusinersen. In addition, the selection of the right chemistry might make a difference at a later stage of the ASO drug development. Drisapersen, which is built on the phosphorothioate backbone with the 2′-OMe modification, was not approved by the FDA due to its failure to meet primary or secondary endpoints as well as severe side effects after local administrations (Goemans et al., 2018). While several chemistries have been utilized in the clinic, direct comparisons of the efficacies and safety of various ASO chemistries and modifications can be difficult to make between studies due to differences in ASO synthesis, animal models used, and dose administration procedures. A study by Sheng et al. (2020) directly compared the efficacies of the PMO and 2′-MOE modifications, which are both FDA approved chemistries for splice-switching ASOs. Equimolar doses of either PMO or 2′-MOE ASOs were injected subcutaneously into the upper backs of severe neonatal SMA mouse models to evaluate the extent of SMN2 exon 7 inclusion. It was found that both chemistries were able to correct *SMN2* splicing and rescue motor function in SMA mice. While the 2′-MOE modification conferred greater and persistent therapeutic benefits, with longer lifespans, increased body-weight gain, and greater retention of motor neurons; the neutrally charged PMO was able to permeate the blood-brain barrier more efficiently than the 2′-MOE, likely because of the latter’s negatively charged phosphorothioate backbone. In addition, the PMOs have shown a good safety profile to date and could be used at much higher doses than the phosphorothioate based ASOs. The FDA had granted Sarepta Therapeutics approval to increase PMO Eteplirsen doses to 100 mg/kg if necessary, whereas the phosphorothioate, 2′-OMe Drisapersen could not be applied at doses higher than 6 mg/kg without serious adverse effects.

While validating ASO candidates with optimal chemistry provides proof-of-concept evidence of potential clinical benefits, preclinical animal studies are normally required to increase the chances of Phase 1 clinical trial success by giving more information on the delivery route, dosage and dose frequency, while allowing monitoring for possible off-target reactions (Kempf et al., 2018). However, it is well recognized that animal models of human diseases frequently do not reflect the conditions in humans. The *mdx* mouse model of dystrophinopathy initially used to develop and refine the dystrophin exon skipping applications for Duchenne MD does not obviously reflect the severity of the human condition. Although the consequences of dystrophin loss can be seen with histological and immunohistochemical testing, the *mdx* mice live a near normal life span, with symptoms from the dystrophin loss becoming phenotypically obvious later in life, unless the *mdx* mice were crossed with utrophin deficient animals to generate the very severely affected dys*^–/–^*, utr^–/–^ *dko* mouse (Deconinck et al., 1997). Conversely, the first *Smn* knock-out mice were found to be embryonic lethal, as the *SMN2* duplication occurred in the primate lineage and mice did not carry *Smn2* (Schrank et al., 1997). The complete loss of functional SMN protein is incompatible with life, and it was necessary to first “humanize” mice with *SMN2* copies before the *Smn* gene could be deleted to generate SMA mouse models (Hsieh-Li et al., 2000; Bebee et al., 2012). Although the CRISPR/Cas gene editing technology has greatly accelerated the development of animal models, the time, cost, and relevance of producing an animal model, especially for unique mutations could be questioned and would have to be carefully justified. The FDA Modernization Act 2.0 has cleared the way for alternative models including advanced human cells, organoids and artificial intelligence/machine learning approaches to support the preclinical data pipeline and to reduce the dependence on animal models (Zushin et al., 2023).

Furthermore, human studies for rare disease drug candidates still face additional challenges, including low patient numbers, heterogeneous stages of disease progression, as well as the geographic dispersal of patient populations and uncertainty concerning trial endpoints (Kempf et al., 2018). If clinical trials of novel targeted therapies for rare disease are to be meaningful, the selective recruitment of patients with amenable mutations is essential, along with an improved and efficient diagnostic capacity. Unfortunately, this would further reduce the potential number of trial participants, which again raises the small patient cohort challenge for clinical trial design and might be a different situation to the new small-cohort clinical trial design paradigm introduced following the approval of “N-of-1” studies such as that of Milasen. Additionally, alternative statistical methods ideal for such trials are now being investigated by the Small Population Clinical Trials Task Force, in conjunction with governing agencies (Day et al., 2018). Because of the geographically dispersed nature of patients with rare conditions, it is essential to facilitate multi-institute, multi-nation and multi-stakeholder initiatives for more sustainable and affordable orphan drug development (Boycott et al., 2019). However, it is important to distinguish between rare diseases, such as Duchenne MD and SMA, and ultra-rare diseases, which has been defined by the n-Lorem charity as patients having an N-of-1 condition, or a having a disease that affects < 30 patients worldwide (Crooke, 2021). Ultra-rare diseases are more likely to receive accelerated regulatory approval compared to rare diseases because treatment approaches for ultra-rare conditions are often investigational, focusing on exploring new treatments rather than developing universally safe and effective solutions. On the other hand, rare diseases will have to endure the traditional drug approval procedure involving multi-phase clinical trials, and rigorous evaluations of drug safety and efficacy for the general population.

With the advancements in technologies, we are better equipped to understand disease pathogenesis and identify mutations potentially amenable to antisense therapeutic strategies. ASO chemistries and modifications may vary in their mechanisms of action, however similarities in toxicology and pharmacokinetic profiles can somewhat streamline the drug-discovery process. With improvements in chemistry and synthesizer technology, high quality oligonucleotides can now be produced faster and at lower cost than ever before (Hughes and Ellington, 2017). Although manufacturing expenses for clinical grade antisense compounds are considerable, this does not necessarily correlate with the final price of FDA approved drugs. For example, the cost to synthesize sufficient Spinraza for one SMA patient for a year has been estimated not to be substantial. However, patients are charged ∼$750,000 USD for the four dosages required in the first year of treatment, which is then reduced by around half for maintenance doses in subsequent years (Rodrigues and Yokota, 2018; Wang et al., 2022; Starner and Gleason, 2019). The amount charged by pharmaceutical companies aims to recoup expenses attributed to drug development, failed compounds, clinical trials costs and future research (Annett, 2021). Thus, to make antisense therapeutics accessible to patients, new models and clinical trial protocols are needed to increase commercial viability (Crooke, 2021; Crooke et al., 2021b).

Overcoming the challenges in animal modeling for rare diseases, improving clinical trial design for small patient numbers, and developing antisense therapeutic strategies to treat a broader range of diseases, will make a substantial contribution to novel drug discovery for rare conditions, leading to positive outcomes for patients and families affected by these diseases.
